# Development and validation of an interpretable risk prediction model for perioperative ischemic stroke in noncardiac, nonvascular, and nonneurosurgical patients: a retrospective study

**DOI:** 10.3389/fphys.2025.1628475

**Published:** 2025-07-30

**Authors:** Xuhui Cong, Xuli Zou, Ruilou Zhu, Yubao Li, Lu Liu, Jiaqiang Zhang

**Affiliations:** ^1^ Department of Anesthesia and Perioperative Medicine, Zhengzhou University People’s Hospital and Henan Provincial People’s Hospital, Zhengzhou, Henan, China; ^2^ Xinxiang Medical University, Xinxiang, Henan, China; ^3^ Zhengzhou University, Zhengzhou, Henan, China

**Keywords:** prediction model, risk assessment, perioperative stroke, noncardiac, nonvascular, and nonneurosurgical procedures, general anesthesia

## Abstract

**Background:**

Perioperative stroke is a severe complication for patients undergoing non-cardiac, non-vascular, and non-neurosurgical surgeries, resulting in significant morbidity and mortality. Despite its clinical relevance, effective predictive models for stroke risk in this population are scarce. This study seeks to develop and validate an interpretable predictive model that incorporates essential perioperative variables to assess stroke risk. The goal is to enhance risk stratification and support more informed clinical decision-making.

**Methods:**

A retrospective cohort study included 106,328 patients aged 18 years or older who underwent non-cardiac, non-vascular, and non-neurosurgical surgeries at our institution. The development cohort comprised 74,429 patients, with 140 perioperative stroke incidents, while the validation cohort consisted of 31,899 patients, with 59 incidents. Risk factors for perioperative stroke were identified using univariable logistic regression analysis. The Least Absolute Shrinkage and Selection Operator (LASSO) regression method was applied to select variables, followed by the development, validation, and performance evaluation of the predictive model using multivariate logistic regression analysis.

**Results:**

The prediction model, developed using nine variables including demographic information, medical history, and pre- and post-operative data, demonstrated strong discriminatory power in predicting perioperative stroke (AUC = 0.869; 95% CI, 0.827–0.910). It also exhibited an excellent fit with the validation cohort (Hosmer–Lemeshow test, χ^2^ = 6.877, *P* = 0.650). Additionally, the SHAP (Shapley Additive Explanations) interpretability model was integrated to enhance the model’s transparency, allowing clinicians to better understand the contribution of each predictor. Decision curve analysis confirmed the model’s significant net benefit, further validating its clinical utility.

**Conclusion:**

This study developed and validated a robust predictive model for perioperative stroke risk in patients undergoing non-cardiac, non-vascular, and non-neurosurgical procedures. Despite its retrospective design, the model exhibited strong performance and clinical relevance. It provides a solid foundation for future multi-center studies aimed at refining and expanding its applicability.

## Introduction

Perioperative stroke is a serious complication that can occur within 30 days after surgery, leading to significant neurological dysfunction, prolonged hospitalization, and increased mortality rates (Vlisides and Mashour, 2016). The incidence of perioperative stroke varies across different surgery types, ranging from 0.1% to 1.9% in non-cardiac surgeries to 1.9%–9.7% in cardiovascular surgeries (Ng et al., 2011; Marcucci et al., 2023). While several risk factors for perioperative stroke have been identified, traditional statistical-based prediction models struggle with nonlinearity and variable selection issues (Chahine et al., 2023). Recently, machine learning has emerged as a promising tool for predicting perioperative stroke in patients undergoing non-cardiac surgeries (Fernandes et al., 2021; Abraham et al., 2023).

This study aims to collect preoperative and intraoperative data, including relevant variables and demographics, from patients undergoing non-cardiac surgeries to develop an interpretable risk prediction model for perioperative stroke. The results will enhance physicians’ understanding of the mechanisms behind perioperative stroke, ultimately supporting informed decision-making and improving stroke prevention and prediction.

## Materials and methods

### Study design and study population

Data from the electronic medical records of patients treated at Henan Provincial People’s Hospital between November 2014 and June 2021 were retrospectively analyzed. This period was selected because it provides the most consistent dataset from our electronic medical record (EMR) database, which was established in 2021. Although the database extends to 2023, advancements in surgical techniques and perioperative protocols after 2021 could introduce variability, so the analysis was limited to this timeframe for consistency. Ethical approval for the study was granted by the Ethics Review Committee of Henan Provincial People’s Hospital (Approval No. 2021-157), and all methods adhered to the Declaration of Helsinki. Informed consent was waived by the committee, as the study complied with relevant ethical guidelines and regulations.

**FIGURE 1:**
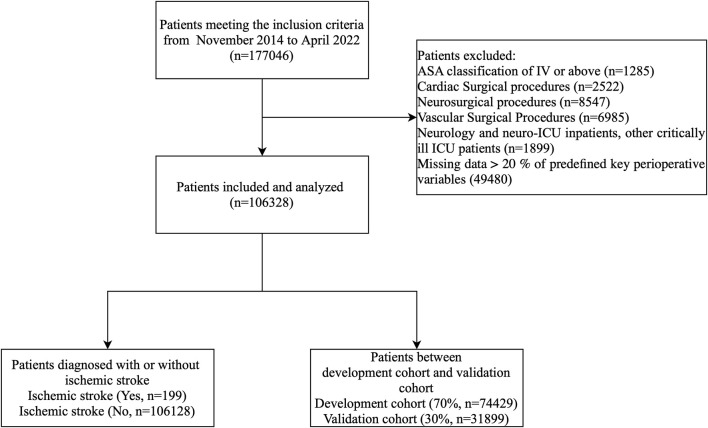
Patient flow diagram. ASA, American Society of Anesthesiologists.

Patients aged ≥18 years who underwent any surgical procedure under general anesthesia at Henan Provincial People’s Hospital between November 1, 2014, and June 30, 2021 (n = 177,046) were identified from our electronic medical record system. To ensure comprehensive data capture, patients included in the study were required to have at least one follow-up within 30 days after surgery. Follow-up visits were scheduled to capture the occurrence of perioperative strokes. However, in cases where patients did not have a stroke during their follow-up visit but had one after the visit but still within 30 days, additional monitoring through the hospital’s EHR system was used to capture these events. Any stroke occurring after a follow-up visit within the 30-day period was recorded, and these patients were included in the analysis. We applied the following exclusion criteria in a stepwise fashion (Figure 1).

1. American Society of Anesthesiologists physical status IV or V (n = 1,285);
2. Cardiac surgical procedures (n = 2,522);
3. Neurosurgical procedures (n = 8,547);
4. Vascular surgical procedures (n = 6,985);
5. Admission to neurology wards, neuro–intensive care units, or other critically ill ICUs (n = 1899);
6. Missing data >20% of predefined key perioperative variables (n = 49,480).

After sequential exclusions, 106,328 patients remained for analysis. Within this analytic cohort, 199 patients experienced a perioperative ischemic stroke and 106,129 did not. To develop and validate the risk prediction model, we randomly allocated 70% of the cohort (n = 74,429) to the development cohort and the remaining 30% (n = 31,899) to the validation cohort, preserving the overall event rate.

### Data preprocessing

Missing values were handled using multiple imputation with the MICE (Multivariate Imputation by Chained Equations) package in R. This method generates multiple plausible values for each missing data point, resulting in several complete datasets. The results from these datasets were combined to provide robust estimates while accounting for the uncertainty of missing data. Sensitivity analyses were performed to assess the impact of imputation on the model’s performance.

### Variables

This study included clinical data from 106,328 patients who underwent non-cardiac, non-vascular surgeries. Perioperative demographic, clinical, imaging, and laboratory data were systematically collected from the preoperative period through the first 7 days post-surgery. The study focused on the following variables.

1. The demographic characteristics included age, sex, height, weight, and BMI (Body Mass Index).
2. The surgery-related factors, such as ASA (American Standards Association) grade, type and duration of surgery, anaesthesia duration and method, urine output, and blood loss volume, were also included.
3. The comorbidities included a history of smoking and alcohol consumption, ascites, hypertension, diabetes mellitus, various cardiac conditions, COPD (chronic obstructive pulmonary disease), renal issues, and cerebrovascular diseases.
4. The laboratory test results included blood counts; serum creatinine levels; albumin levels; liver enzymes; and various coagulation profiles.
5. Preoperative medications included a range of antihypertensive, anticoagulant, antiplatelet, and other drugs, along with specific details such as the duration of discontinuation of certain medications.
6. The vital signs monitored intraoperatively included blood pressure, heart rate, temperature, BIS (bispectral index) value, and end-tidal carbon dioxide.
7. Intraoperative medications ranged from inhaled anaesthetics to various drugs, including diuretics and anticoagulants.
8. Intraoperative fluids and blood transfusions included colloids, crystalloids, and various blood components.
9. Intraoperative vasopressor drugs encompassed a range of medications from ephedrine to phenylephrine.
10. Postoperative medications included statins, anticoagulants, antiplatelets, and other drugs, along with details regarding their dosages and durations.

Postoperative medications, including anticoagulants, antiplatelet drugs, and statins, were administered during the first 7 days after surgery to prevent secondary cardiovascular or cerebrovascular events.

### Perioperative ischemic stroke definition

Perioperative ischemic stroke was defined as a new focal neurological deficit occurring within 30 days after surgery, confirmed by neuroimaging (CT or MRI), and diagnosed by board-certified neurologists (Mashour et al., 2011). Diagnoses were recorded in the patients’ medical records. To account for strokes that occurred after follow-up visits but still within the 30-day period, we employed a robust data collection approach using the hospital’s electronic health record (EHR) system to capture any stroke events. Additionally, patients were monitored for adverse events through follow-up calls and subsequent clinic visits.

### Statistical analysis

To ensure adequate statistical power, a power calculation was conducted prior to the study. Based on an estimated perioperative stroke incidence of 0.19% (consistent with previous studies) and the requirement of at least 10 events per variable (EPV: Events Per Variable) for logistic regression, a minimum of 160 stroke events was needed to analyze 16 predictor variables. Our cohort included 199 stroke events, exceeding this threshold and providing sufficient power (>80%) to detect significant predictors with an alpha level of 0.05. The total cohort size of 106,328 patients ensured adequate stroke event capture while maintaining a representative sample.

Various statistical methods were used to analyze continuous and categorical variables. Continuous variables are presented as medians with interquartile ranges (25th to 75th percentiles), with differences assessed using the Mann–Whitney U test. Categorical variables are presented as frequencies and percentages, with differences evaluated using the chi-square test or Fisher’s exact test.

Logistic regression analysis was performed using the glmnet package in R, with the LASSO (Least Absolute Shrinkage and Selection Operator) method applied for variable selection. Hyperparameter tuning for LASSO logistic regression was performed via tenfold cross-validation, with the optimal lambda value selected based on the minimum cross-validation error. Significant variables identified by LASSO were further incorporated into the logistic regression model using a forward stepwise selection procedure to minimize the Akaike information criterion (AIC), ensuring a parsimonious model with minimal variable inclusion.

Multivariate logistic regression analysis was used to assess multicollinearity by calculating the variance inflation factor (VIF). VIF values below 10 indicated no multicollinearity concerns. For variables significantly correlated with the main outcomes, odds ratios (ORs) and 95% confidence intervals (CIs) were calculated.

The model’s discriminative ability was evaluated using a diagnostic chart that included key variables. Discriminative ability was assessed with the receiver operating characteristic (ROC) curve, with the area under the curve (AUC) quantifying model performance. Calibration was assessed using calibration plots and Spiegelhalter’s Z test, which indicated good calibration when predicted values closely matched actual risk. Additionally, the SHAP (Shapley Additive Explanations) interpretability model was applied to provide insights into the contribution of each predictor, enhancing the model’s transparency and interpretability for clinical decision-making.

A randomly generated sample of 70% of the cohort (using a seed) was applied for the development cohort, with the remaining 30% used for the validation cohort. The rms package in R was employed for graphical evaluation. All tests were two-sided, with a p-value of less than 0.05 indicating statistical significance. Statistical analysis was conducted using R version 4.3.1.

## Results

A total of 106,328 adult patients met the inclusion criteria during the study period, and after applying the exclusion criteria, the study cohort consisted of 70,718 patients. We analyzed data from 199 perioperative stroke patients, allocating 140 to the development cohort and 59 to the validation cohort. Tables 1,2 present descriptive statistical analyses: Table 1 compares patients with and without ischemic stroke, while Table 2 contrasts the development and validation cohorts.

**TABLE 1:** Baseline demographic and clinical characteristics of included patients diagnosed with or without ischemic stroke.

Variables	Categories	Total (n = 106,328)	No postoperative ischemic stroke	Postoperative ischemic stroke	*P*
			No (n = 106,129)	Yes (n = 199)	
Emergency operation, n (%)					<0.001
	No	92,972 (87.4)	92,818 (87.4)	154 (77.4)	
	Yes	13,356 (12.6)	13,311 (12.5)	45 (22.6)	
Sex, n (%)					<0.001
	male	46,481 (43.7)	46,369 (43.7)	112 (56.3)	
	female	59,847 (56.3)	59,760 (56.3)	87 (43.7)	
Age, (Median [Q1, Q3]), yr		52 (40, 63)	52 (40, 63)	68 (62, 75)	<0.001
Duration of the procedure, (Median [Q1, Q3]), min		140 (100, 210)	140 (100, 210)	170 (120, 245)	<0.001
Duration of anesthesia, (Median [Q1, Q3]), min		156 (110, 230)	155 (110, 230)	190 (133, 265)	<0.001
ASA classification, n (%)					<0.001
	I	10,816 (10.2)	10,816 (10.2)	0 (0.0)	
	II	79,995 (75.3)	79,897 (75.3)	98 (49.2)	
	III	15,517 (14.6)	15,416 (14.5)	101 (50.8)	
Methods of anesthesia, n (%)					0.106
	Simple general anesthesia	72,209 (67.9)	72,085 (67.9)	124 (62.3)	
	General anesthesia combined with nerve block	34,119 (32.1)	34,044 (32.1)	75 (37.7)	
Urine output, (Median [Q1, Q3]), ml		300 (100, 500)	300 (100, 500)	300 (200, 500)	0.115
Amount of blood loss, (Median [Q1, Q3]), ml		50 (20, 200)	50 (20, 200)	100 (30, 200)	<0.001
History of smoking, n (%)					0.881
	No	97,817 (92.0)	97,635 (92.0)	182 (91.5)	
	Yes	8,511 (8.0)	8,494 (8.0)	17 (8.5)	
History of drinking, n (%)					0.002
	No	95,825 (90.1)	95,659 (90.1)	166 (83.4)	
	Yes	10,503 (9.9)	10,470 (9.9)	33 (16.6)	
Ascites, n (%)					1
	No	101,938 (95.9)	101,747 (95.9)	191 (96.0)	
	Yes	4,390 (4.1)	4,382 (4.1)	8 (4.0)	
Hypertension, n (%)					<0.001
	No	93,361 (87.8)	93,227 (87.8)	134 (67.3)	
	Yes	12,967 (12.2)	12,902 (12.2)	65 (32.7)	
Diabetes, n (%)					<0.001
	No	99,138 (93.2)	98,983 (93.2)	155 (77.9)	
	Yes	7,190 (6.8)	7,146 (6.8)	44 (22.1)	
Coronary heart disease, n (%)					<0.001
	No	102,624 (96.5)	102,448 (96.5)	176 (88.4)	
	Yes	3,704 (3.5)	3,681 (3.5)	23 (11.6)	
Stenocardia, n (%)					<0.001
	No	106,155 (99.8)	105,961 (99.8)	194 (97.5)	
	Yes	173 (0.2)	168 (0.2)	5 (2.5)	
Valvular heart disease, n (%)					0.014
	No	105,583 (99.3)	105,389 (99.3)	194 (97.5)	
	Yes	745 (0.7)	740 (0.7)	5 (2.5)	
Myocardial infarction, n (%)					<0.001
	No	105,968 (99.7)	105,776 (99.7)	192 (96.5)	
	Yes	360 (0.3)	353 (0.3)	7 (3.5)	
Heart failure, n (%)					0.02
	No	106,214 (99.0)	106,017 (99.9)	197 (99.0)	
	Yes	114 (1.0)	112 (0.1)	2 (1.0)	
Arrhythmia, n (%)					0.132
	No	105,290 (99.0)	105,095 (99.0)	195 (98.0)	
	Yes	1,038 (1.0)	1,034 (1.0)	4 (2.0)	
Atrial fibrillation, n (%)					0.134
	No	105,987 (99.7)	105,790 (99.7)	197 (99.0)	
	Yes	341 (0.3)	339 (0.3)	2 (1.0)	
Intracoronary stent implantation, n (%)					0.027
	No	105,728 (99.4)	105,533 (99.4)	195 (98.0)	
	Yes	600 (0.6)	596 (0.6)	4 (2.0)	
Cardiac surgery, n (%)					<0.001
	No	13,284 (12.5)	13,284 (12.5)	0 (0)	
	Yes	93,044 (87.5)	92,845 (87.5)	199 (100.0)	
Peripheral vascular disease, n (%)					0.013
	No	99,429 (93.5)	99,252 (93.5)	177 (88.9)	
	Yes	6,899 (6.5)	6,877 (6.5)	22 (11.1)	
COPD, n (%)					1
	No	105,543 (99.3)	105,345 (99.3)	198 (99.5)	
	Yes	785 (0.7)	784 (0.7)	1 (0.5)	
Dialysis, n (%)					0.594
	No	104,364 (98.2)	104,167 (98.2)	197 (99.0)	
	Yes	1964 (1.8)	1962 (1.8)	2 (1.0)	
Renal insufficiency, n (%)					0.638
	No	105,685 (99.4)	105,486 (99.4)	199 (100.0)	
	Yes	643 (0.6)	643 (0.6)	0 (0.0)	
History of cerebrovascular disease, n (%)					<0.001
	No	102,270 (96.2)	102,128 (96.2)	142 (71.4)	
	Yes	4,058 (3.8)	4,001 (3.8)	57 (28.6)	
TIA, n (%)					1
	No	106,261 (100.0)	106,062 (100.0)	199 (100)	
	Yes	67 (0.0)	67 (0.0)	0 (0)	
Stroke, n (%)					<0.001
	No	102,725 (96.6)	102,579 (96.6)	146 (73.4)	
	Yes	3,603 (3.4)	3,550 (3.4)	53 (26.6)	
Paraplegia, n (%)					<0.001
	No	106,134 (99.8)	105,939 (99.8)	195 (98.0)	
	Yes	194 (0.2)	190 (0.2)	4 (2.0)	
Cancer, n (%)					0.128
	No	93,815 (88.2)	93,632 (88.2)	183 (92.0)	
	Yes	12,513 (11.8)	12,497 (11.8)	16 (8.0)	
Preoperative white blood cell count, (Median [Q1, Q3]), 10^9^/L		6.35 (5.1, 8.2)	6.35 (5.1, 8.2)	6.73 (5.16, 8.54)	0.139
Preoperative red blood cell count, (Median [Q1, Q3]), 10^12^/L		4.21 (3.8, 4.61)	4.22 (3.8, 4.61)	3.99 (3.54, 4.4)	<0.001
Preoperative platelet count, (Median [Q1, Q3]), 10^9^/L		226 (183, 274)	226 (183, 274)	215 (165.5, 271)	0.04
Preoperative hemoglobin levels, (Median [Q1, Q3]), g/L		126 (112, 139)	126 (112, 139)	120 (106.5, 134)	<0.001
Preoperative serum creatinine, (Median [Q1, Q3]), μmol/L		55 (47, 66)	55 (47, 66)	56 (48, 72)	0.024
Preoperative serum albumin levels, (Median [Q1, Q3]), g/L		40.7 (37, 44)	40.7 (37, 44)	37 (34.05, 40.5)	<0.001
Preoperative alanine aminotransferase, (Median [Q1, Q3]), U/L		18 (12.6, 29)	18 (12.6, 29)	18 (12.5, 31)	0.681
Preoperative aspartate aminotransferase, (Median [Q1, Q3]), U/L		19.4 (16, 25.7)	19.4 (16, 25.7)	21.5 (16.65, 30)	0.01
Preoperative serum sodium, (Median [Q1, Q3]), mmol/L		141 (139, 142)	141 (139, 142)	140.7 (138, 142)	0.013
Preoperative blood potassium, (Median [Q1, Q3]), mmol/L		4.17 (3.9, 4.43)	4.17 (3.9, 4.43)	4.09 (3.74, 4.38)	0.001
Preoperative blood calcium, (Median [Q1, Q3]), mmol/L		2.24 (2.14, 2.33)	2.24 (2.14, 2.33)	2.18 (2.08, 2.28)	<0.001
Preoperative thrombin time, (Median [Q1, Q3]), s		16.5 (15.5, 17.6)	16.5 (15.5, 17.6)	16.6 (15.6, 17.5)	0.644
Preoperative plasma activated partial thromboplastin time, (Median [Q1, Q3]), s		34.1 (30.4, 37.5)	34.1 (30.4, 37.5)	34.1 (29.65, 37.5)	0.905
Preoperative plasma prothrombin time, (Median [Q1, Q3]), s		12.1 (11.5, 12.9)	12.1 (11.5, 12.9)	12.3 (11.4, 13.1)	0.059
Preoperative international normalized ratio, (Median [Q1, Q3])		0.93 (0.87, 1.02)	0.93 (0.87, 1.02)	0.95 (0.88, 1.08)	0.006
Intraoperative MAP ≤60 mmHg for 5–10 min, n (%)					<0.001
	No	105,195 (99.0)	105,006 (99.0)	189 (95.0)	
	Yes	1,133 (1.0)	1,123 (1.0)	10 (5.0)	
Preoperative ACEI drugs, n (%)					<0.001
	No	100,054 (94.1)	99,884 (94.1)	170 (85.4)	
	Yes	6,274 (5.9)	6,245 (5.9)	29 (14.6)	
Perioperative non-steroidal drugs, n (%)					<0.001
	No	22,707 (21.4)	22,700 (21.4)	7 (3.5)	
	Yes	83,621 (78.6)	83,429 (78.6)	192 (96.5)	
Postoperative intravenous thrombolysis, n (%)					0.033
	No	106,310 (100.0)	106,112 (100.0)	198 (99.5)	
	Yes	18 (0.0)	17 (0.0)	1 (0.5)	
Postoperative statins, n (%)					<0.001
	No	104,115 (97.9)	103,985 (97.9)	130 (65.3)	
	Yes	2,213 (2.1)	2,144 (2.1)	69 (34.7)	
Postoperative statins, n (%)					<0.001
	No	104,115 (98)	103,985 (98)	130 (65)	
	Yes	2,213 (2)	2,144 (2)	69 (35)	
Postoperative anticoagulants, n (%)					<0.001
	No	71,861 (67.6)	71,815 (67.7)	46 (23.1)	
	Yes	34,467 (32.4)	34,314 (32.3)	153 (76.9)	
Postoperative antiplatelet drugs, n (%)					<0.001
	No	102,252 (96.3)	102,126 (96.2)	126 (63.3)	
	Yes	4,076 (3.8)	4,003 (3.8)	73 (36.7)	
Postoperative butylphthalides, n (%)					<0.001
	No	106,102 (99.8)	105,965 (99.8)	137 (68.8)	
	Yes	226 (0.2)	164 (0.2)	62 (31.2)	

Abbreviations: ASA, american society of anesthesiologists; COPD, chronic obstructive pulmonary disease; TIA, transient ischemic attack.

**TABLE 2:** Baseline demographic and clinical characteristics of included patients between training cohort and validation cohort.

Variables	Categories	Total (n = 106,328)	Training cohort (n = 74,429)	Validation cohort (n = 31,899)	*P*
Ischemic stroke, n (%)					0.975
	No	106,129 (100.0)	74,289 (100.0)	31,840 (100.0)	
	Yes	199 (0.0)	140 (0.0)	59 (0.0)	
Emergency operation, n (%)					0.894
	No	92,972 (87.4)	65,087 (87.6)	27,885 (87.6)	
	Yes	13,356 (12.6)	9,342 (12.6)	4,014 (12.6)	
Sex, n (%)					0.572
	male	46,481 (43.7)	32,494 (43.7)	13,987 (43.9)	
	female	59,847 (56.3)	41,935 (56.4)	17,912 (56.2)	
Age, (Median [Q1, Q3]), yr		52 (40, 63)	52 (40, 63)	52 (40, 63)	0.316
Duration of the procedure, (Median [Q1, Q3]), min		140 (100, 210)	140 (100, 210)	140 (100, 210)	0.56
Duration of anesthesia, (Median [Q1, Q3]), min		156 (110, 230)	156 (110, 230)	155 (110, 230)	0.582
ASA classification, n (%)					0.875
	I	10,816 (10.2)	7,579 (10.2)	3,237 (10.2)	
	II	79,995 (75.3)	55,964 (75.4)	24,031 (75.5)	
	III	15,517 (14.6)	10,886 (14.6)	4,631 (14.5)	
Methods of anesthesia, n (%)					0.47
	Simple general anesthesia	72,209 (67.9)	50,495 (68.0)	21,714 (68.2)	
	General anesthesia combined with nerve block	34,119 (32.1)	23,934 (32.0)	10,185 (32.0)	
Urine output, (Median [Q1, Q3]), ml		300 (100, 500)	300 (100, 500)	300 (100, 500)	0.938
Amount of blood loss, (Median [Q1, Q3]), ml		50 (20, 200)	50 (20, 200)	50 (20, 200)	0.128
History of smoking, n (%)					0.508
	No	97,817 (92.0)	68,444 (92.0)	29,373 (92.0)	
	Yes	8,511 (8.0)	5,985 (8.0)	2,526 (8.0)	
History of drinking, n (%)					0.388
	No	95,825 (90.0)	67,038 (90.1)	28,787 (89.9)	
	Yes	10,503 (10.0)	7,391 (9.9)	3,112 (10.1)	
Ascites, n (%)					0.698
	No	101,938 (96.0)	71,344 (95.9)	30,594 (96.2)	
	Yes	4,390 (4.0)	3,085 (4.1)	1,305 (3.8)	
Hypertension, n (%)					0.859
	No	93,361 (87.9)	65,343 (87.9)	28,018 (87.9)	
	Yes	12,967 (12.1)	9,086 (12.1)	3,881 (12.1)	
Diabetes, n (%)					0.416
	No	99,138 (93.2)	69,365 (93.2)	29,773 (93.2)	
	Yes	7,190 (6.8)	5,064 (6.8)	2,126 (6.8)	
Coronary heart disease, n (%)					0.367
	No	102,624 (96.4)	71,811 (96.5)	30,813 (96.3)	
	Yes	3,704 (3.6)	2,618 (3.5)	1,086 (3.7)	
Stenocardia, n (%)					0.816
	No	106,155 (99.8)	74,306 (99.8)	31,849 (99.8)	
	Yes	173 (0.2)	123 (0.2)	50 (0.2)	
Valvular heart disease, n (%)					1
	No	105,583 (99.3)	73,907 (99.3)	31,676 (99.3)	
	Yes	745 (0.7)	522 (0.7)	223 (0.7)	
Myocardial infarction, n (%)					0.454
	No	105,968 (99.7)	74,170 (99.7)	31,798 (99.7)	
	Yes	360 (0.3)	259 (0.3)	101 (0.3)	
Heart failure, n (%)					0.728
	No	106,214 (99.9)	74,347 (99.9)	31,867 (99.9)	
	Yes	114 (0.1)	82 (0.1)	32 (0.1)	
Arrhythmia, n (%)					0.245
	No	105,290 (99.0)	73,720 (99.0)	31,570 (99.0)	
	Yes	1,038 (1.0)	709 (1.0)	329 (1.0)	
Atrial fibrillation, n (%)					0.332
	No	105,987 (100.0)	74,199 (100.0)	31,788 (100.0)	
	Yes	341 (0.0)	230 (0.0)	111 (0.0)	
Intracoronary stent implantation, n (%)					0.687
	No	105,728 (99.4)	74,004 (99.4)	31,724 (99.4)	
	Yes	600 (0.6)	425 (0.6)	175 (0.6)	
Cardiac surgery, n (%)					0.457
	No	13,284 (12.5)	9,336 (12.5)	3,948 (12.4)	
	Yes	93,044 (87.5)	65,093 (87.5)	27,951 (87.6)	
Peripheral vascular disease, n (%)					0.573
	No	99,429 (93.5)	69,621 (93.6)	29,808 (93.4)	
	Yes	6,899 (6.5)	4,808 (6.4)	2091 (6.6)	
COPD, n (%)					0.755
	No	105,543 (99.3)	73,884 (99.3)	31,659 (99.2)	
	Yes	785 (0.7)	545 (0.7)	240 (0.8)	
Dialysis, n (%)					1
	No	104,364 (98.2)	73,054 (98.1)	31,310 (98.2)	
	Yes	1964 (1.8)	1,375 (1.9)	589 (1.8)	
Renal insufficiency, n (%)					0.769
	No	105,685 (99.4)	73,975 (99.4)	31,710 (99.4)	
	Yes	643 (0.6)	454 (0.6)	189 (0.6)	
History of cerebrovascular disease, n (%)					0.574
	No	102,270 (96.2)	71,605 (96.2)	30,665 (96.2)	
	Yes	4,058 (3.8)	2,824 (3.8)	1,234 (3.8)	
TIA, n (%)					0.709
	No	106,261 (99.9)	74,384 (99.9)	31,877 (99.9)	
	Yes	67 (0.1)	45 (0.1)	22 (0.1)	
Stroke, n (%)					0.988
	No	102,725 (97.0)	71,906 (97.0)	30,819 (97.0)	
	Yes	3,603 (3.0)	2,523 (3.0)	1,080 (3.0)	
Paraplegia, n (%)					0.227
	No	106,134 (99.8)	74,285 (99.8)	31,849 (99.8)	
	Yes	194 (0.2)	144 (0.2)	50 (0.2)	
Cancer, n (%)					0.149
	No	93,815 (88.2)	65,600 (88.2)	28,215 (88.2)	
	Yes	12,513 (11.8)	8,829 (11.8)	3,684 (11.8)	
Preoperative white blood cell count, (Median [Q1, Q3]), 10^9^/L		6.35 (5.1, 8.2)	6.36 (5.1, 8.2)	6.32 (5.09, 8.19)	0.136
Preoperative red blood cell count, (Median [Q1, Q3]), 10^12^/L		4.21 (3.8, 4.61)	4.21 (3.8, 4.61)	4.22 (3.8, 4.61)	0.408
Preoperative platelet count, (Median [Q1, Q3]), 10^9^/L		226 (183, 274)	225 (183, 273)	226 (183, 274)	0.173
Preoperative hemoglobin levels, (Median [Q1, Q3]), g/L		126 (112, 139)	126 (112, 139)	126 (112, 139)	0.412
Preoperative serum creatinine, (Median [Q1, Q3]), μmol/L		55 (47, 66)	55 (47, 66)	55 (48, 66)	0.623
Preoperative serum albumin levels, (Median [Q1, Q3]), g/L		40.7 (37, 44)	40.7 (37, 44)	40.7 (37, 44)	0.228
Preoperative alanine aminotransferase, (Median [Q1, Q3]), U/L		18 (12.6, 29)	18 (12.6, 29)	18 (12.8, 29)	0.162
Preoperative aspartate aminotransferase, (Median [Q1, Q3]), U/L		19.4 (16, 25.7)	19.4 (16, 25.7)	19.4 (16, 25.7)	0.875
Preoperative serum sodium, (Median [Q1, Q3]), mmol/L		141 (139, 142)	141 (139, 142)	141 (139, 143)	0.078
Preoperative blood potassium, (Median [Q1, Q3]), mmol/L		4.17 (3.9, 4.43)	4.17 (3.9, 4.43)	4.17 (3.91, 4.43)	0.685
Preoperative blood calcium, (Median [Q1, Q3]), mmol/L		2.24 (2.14, 2.33)	2.24 (2.14, 2.33)	2.24 (2.14, 2.33)	0.736
Preoperative thrombin time, (Median [Q1, Q3]), s		16.5 (15.5, 17.6)	16.5 (15.5, 17.6)	16.5 (15.5, 17.6)	0.588
Preoperative plasma activated partial thromboplastin time, (Median [Q1, Q3]), s		34.1 (30.4, 37.5)	34 (30.4, 37.5)	34.1 (30.4, 37.5)	0.775
Preoperative plasma prothrombin time, (Median [Q1, Q3]), s		12.1 (11.5, 12.9)	12.1 (11.5, 12.9)	12.1 (11.5, 12.9)	0.732
Preoperative international normalized ratio, (Median [Q1, Q3])		0.93 (0.87, 1.02)	0.93 (0.87, 1.02)	0.93 (0.87, 1.02)	0.603
Intraoperative MAP ≤60 mmHg for 5–10 min, n (%)					0.047
	No	105,195 (99.0)	73,605 (99.0)	31,590 (99.0)	
	Yes	1,133 (1.0)	824 (1.0)	309 (1.0)	
Preoperative ACEI drugs, n (%)					0.297
	No	100,054 (94.1)	70,000 (94.0)	30,054 (94.2)	
	Yes	6,274 (5.9)	4,429 (6.0)	1845 (5.8)	
Perioperative non-steroidal drugs, n (%)					0.436
	No	22,707 (21.4)	15,943 (21.4)	6,764 (21.2)	
	Yes	83,621 (78.6)	58,486 (78.6)	25,135 (78.8)	
Postoperative intravenous thrombolysis, n (%)					0.572
	No	106,310 (100.0)	74,418 (100.0)	31,892 (100.0)	
	Yes	18 (0.0)	11 (0.0)	7 (0.0)	
Postoperative statins, n (%)					0.653
	No	104,115 (97.9)	72,890 (97.9)	31,225 (97.8)	
	Yes	2,213 (2.1)	1,539 (2.1)	674 (2.2)	
Postoperative anticoagulants, n (%)					0.393
	No	71,861 (67.6)	50,242 (67.5)	21,619 (67.8)	
	Yes	34,467 (32.4)	24,187 (32.5)	10,280 (32.2)	
Postoperative antiplatelet drugs, n (%)					0.991
	No	102,252 (96.2)	71,575 (96.2)	30,677 (96.2)	
	Yes	4,076 (3.8)	2,854 (3.8)	1,222 (3.8)	
Postoperative butylphthalides, n (%)					0.631
	No	106,102 (99.8)	74,267 (99.8)	31,835 (99.8)	
	Yes	226 (0.2)	162 (0.2)	64 (0.2)	

Abbreviations: ASA, american society of anesthesiologists; COPD, chronic obstructive pulmonary disease; TIA, transient ischemic attack.

**FIGURE 2:**
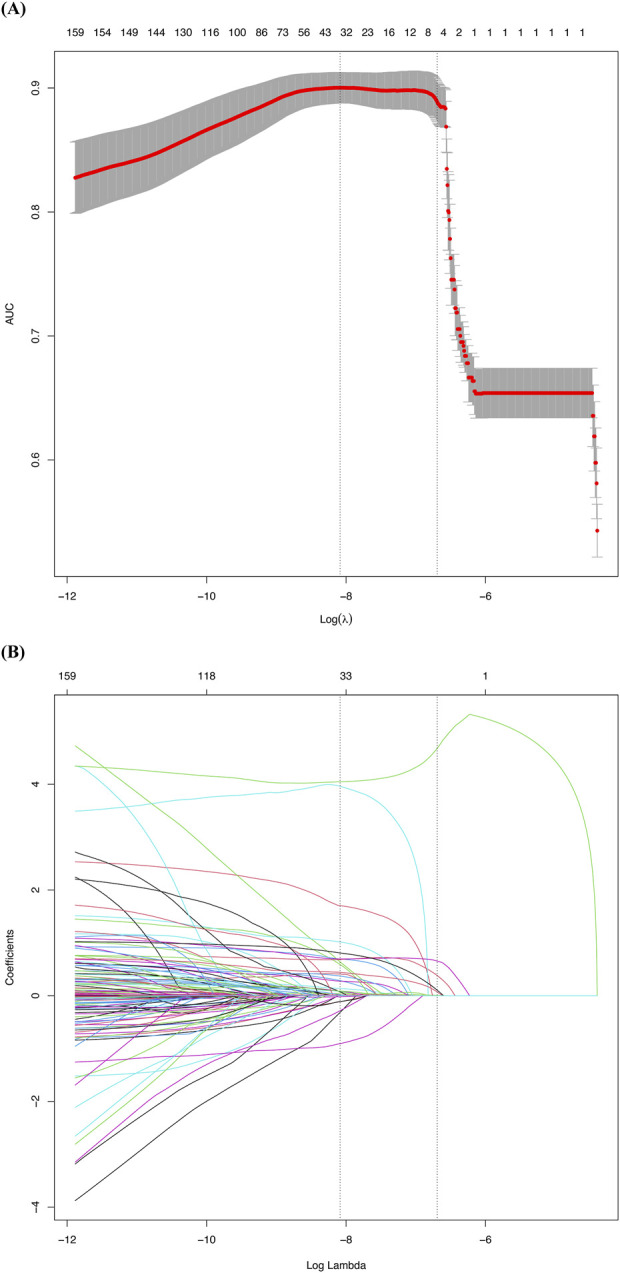
Factor selection using the least absolute shrinkage and selection operator (LASSO) logistic regression: **(A)** The cross-validation curve, where the vertical axis represents the area under the curve (AUC) and the horizontal axis represents log(λ). Two black dashed lines indicate λ.min (the λ value with the highest AUC) and λ.1se (the largest λ within 1 standard error of λ.min). The gray shaded area represents the confidence interval for the mean AUC; **(B)** The LASSO coefficient profiles of the 179 candidate variables. Each curve represents the trajectory of a predictor variable’s coefficient as log(λ) changes. Two black dashed lines indicate λ.min and λ.1se, consistent with Figure 2A.

**FIGURE 3:**
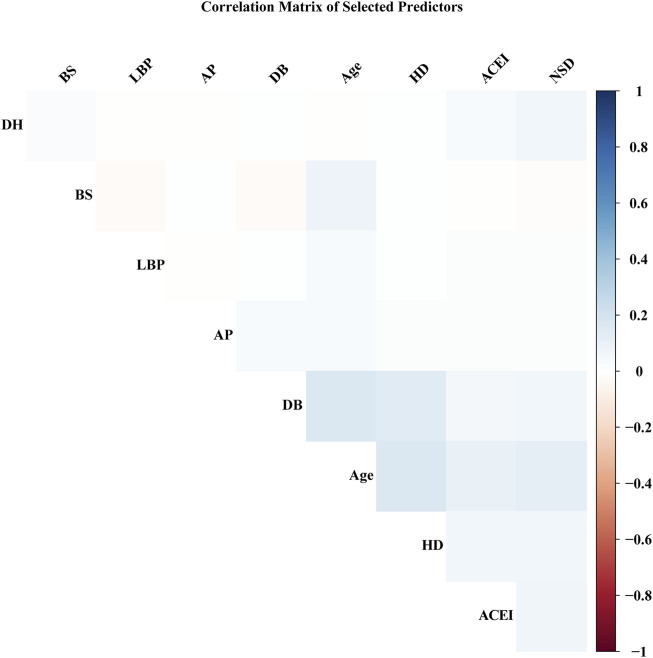
Heatmap of Pairwise Correlations Among Variables. This heatmap visualizes the pairwise correlations between the variables included in the model. Positive correlations are represented in red, while negative correlations are depicted in blue. The intensity of the color reflects the strength of the correlation, with darker shades indicating stronger correlations. HD:history of cerebrovascular disease. DB: diabetes. NSD: perioperative non-steroidal drugs. DH: drinking history. BS: preoperative blood sodium level. ACEI: preoperative ACEI drugs. LBP: intraoperative MAP ≤60 mmHg for 5–10 min. AP: angina pectoris.

To identify predictive factors and develop a corresponding model, we employed the LASSO logistic regression algorithm. Out of 179 factors, 31 with nonzero coefficients were selected based on an optimal lambda value of 0.0003226 (Figures 2A,B). LASSO regression enhances model interpretability by reducing the number of predictors, focusing on the most clinically relevant factors. Figure 3 and Table 3 display the correlation matrix, variance inflation factors (VIF), and tolerance values for each variable, respectively.

**TABLE 3:** Variance inflation factors (VIF) and corresponding tolerance values for each variable.

Variable	VIF	Tolerance
Age	1.07	0.935
Drinking history	1.007	0.993
Diabetes	1.048	0.954
Angina pectoris	1.002	0.998
History of cerebrovascular disease	1.047	0.956
Intraoperative MAP ≤60 mmHg for 5–10 min	1.002	0.998
Preoperative blood sodium level	1.004	0.996
Preoperative ACEI drugs	1.019	0.982

Ultimately, the final model included 9 significant factors: age (OR: 1.07, 95% CI: 1.06–1.09), drinking history (OR: 2.20, 95% CI: 1.44–3.37), diabetes (OR: 1.89, 95% CI: 1.26–2.84), angina pectoris (OR: 8.00, 95% CI: 2.80–22.87), history of cerebrovascular disease (OR: 3.60, 95% CI: 2.42–5.36), intraoperative MAP ≤60 mmHg for 5–10 min (OR: 3.12, 95% CI: 1.42–6.86), preoperative blood sodium level (OR: 0.95, 95% CI: 0.91–0.99), preoperative ACEI drugs (OR: 2.03, 95% CI: 1.31–3.14), perioperative non-steroidal drugs (OR: 4.35, 95% CI: 1.77–10.67) (Table 4).

**TABLE 4:** Multivariate logistic regression analysis for risk factors associated with acute ischemic stroke in perioperative noncardiac, nonvascular, and nonneurosurgical surgery patients.

Variables	B	SE	OR	CI (95%)	Z	*P*
Intercept	−4.762	3.129	0.01	0–3.94	−1.522	0.128
Age	0.068	0.007	1.07	1.06–1.09	9.151	0
Drinking history [Yes]	0.789	0.218	2.2	1.44–3.37	3.621	0
Diabetes [Yes]	0.639	0.206	1.89	1.26–2.84	3.096	0.002
Angina pectoris [Yes]	2.079	0.536	8	2.8–22.87	3.881	0
History of cerebrovascular disease [Yes]	1.282	0.203	3.6	2.42–5.36	6.301	0
Intraoperative MAP ≤60 mmHg for 5–10 min [Yes]	1.138	0.402	3.12	1.42–6.86	2.832	0.005
Preoperative blood sodium level	−0.053	0.022	0.95	0.91–0.99	−2.44	0.015
Preoperative ACEI drugs [Yes]	0.706	0.224	2.03	1.31–3.14	3.152	0.002
Perioperative non-steroidal drugs [Yes]	1.47	0.458	4.35	1.77–10.67	3.212	0.001

Abbreviations: B, regression coefficient; SE, standard error; OR, odds ratio; CI, 95% confidence interval.

**FIGURE 4:**
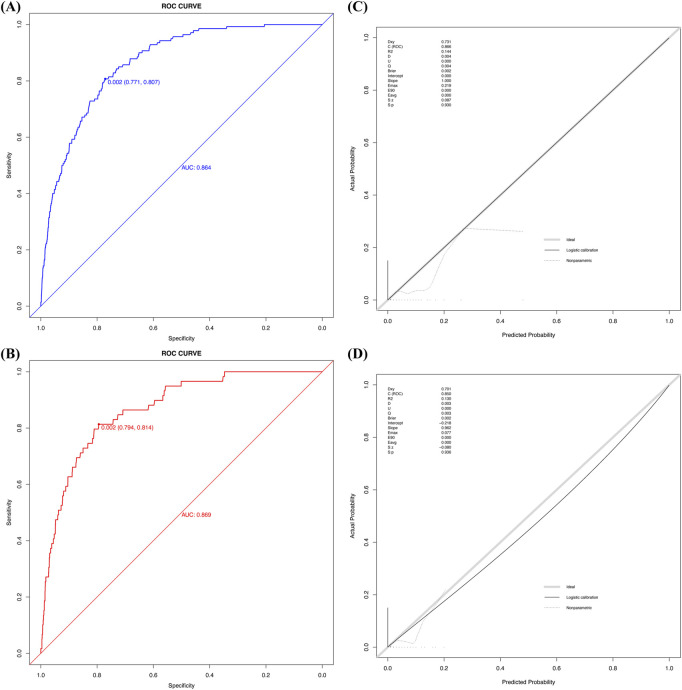
The ROC curve and calibration plot of the predictive model: **(A)** ROC curve for the development cohort, showing an AUC of 0.864 (95% CI: 0.839–0.890), with sensitivity (0.771) and specificity (0.807) at the optimal threshold of 0.002. **(B)** ROC curve for the validation cohort, showing an AUC of 0.869 (95% CI: 0.827–0.910), with sensitivity (0.794) and specificity (0.814) at the same threshold. **(C)** Calibration plot for the development cohort, demonstrating excellent calibration. C-index (0.866): Represents the model’s discriminative ability, similar to AUC. *R* ^2^ (0.144): Indicates the proportion of variance explained by the model. Slope (1) and Intercept (0.000): Reflect perfect calibration, with minimal bias. Brier score (0.002): Measures prediction error, where lower values indicate better calibration. **(D)** Calibration plot for the validation cohort, confirming good calibration. C-index (0.850): Indicates strong discriminative ability. *R* ^2^ (0.130): Reflects the proportion of variance explained. Slope (0.962) and Intercept (−0.218): Highlight a slight deviation from perfect calibration. Brier score (0.002): Confirms low prediction error and good calibration.

After validating the development and validation cohorts, the model achieved an AUC of 0.864 (95% CI 0.839–0.890) in the development cohort and 0.869 (95% CI 0.827–0.910) in the validation cohort (Figures 4A,B). At the optimal threshold of 0.002, sensitivity and specificity were 0.771 and 0.807 in the development cohort, and 0.794 and 0.814 in the validation cohort. The corresponding F1 scores were 0.785 and 0.802, respectively, demonstrating the model’s ability to distinguish between stroke and non-stroke patients despite the imbalanced dataset.

Calibration analysis showed strong agreement between predicted and observed probabilities, with Brier scores of 0.002 in both cohorts. Hosmer–Lemeshow test results indicated good calibration (development cohort: χ^2^ = 5.680, *P* = 0.771; validation cohort: χ^2^ = 6.877, *P* = 0.650). Calibration plots further confirmed the model’s reliability in both cohorts (Figures 4C,D).

**FIGURE 5:**
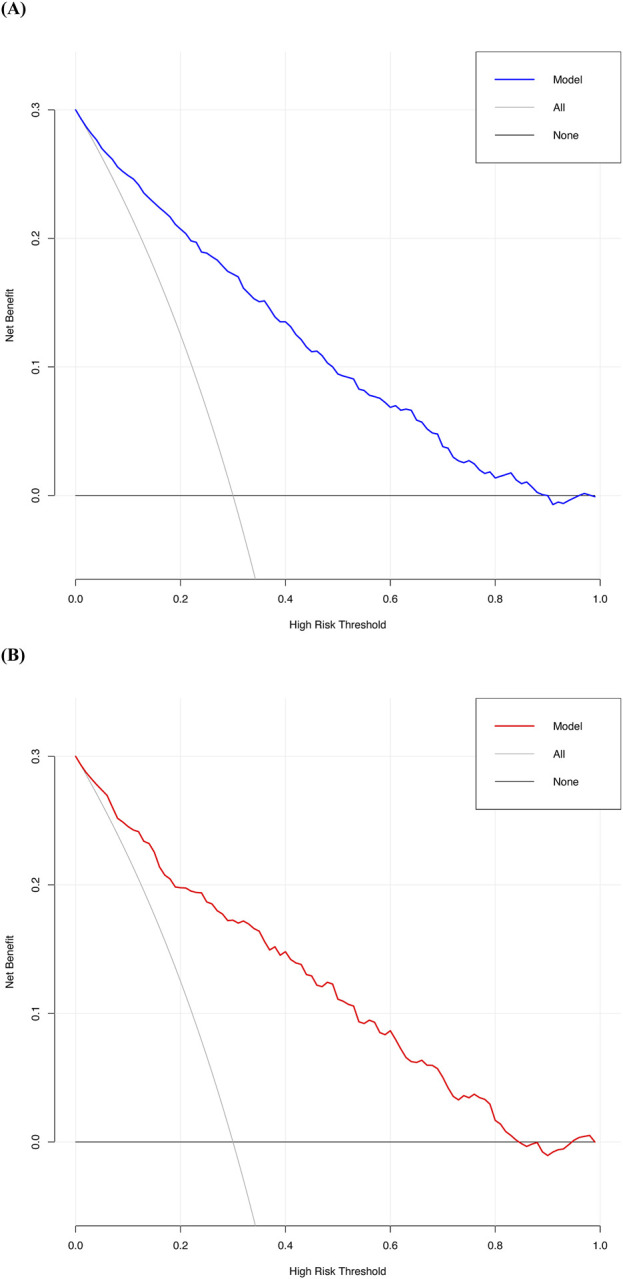
Decision curve analysis for the predictive model: **(A)** Development cohort: The curve illustrates the net benefit of the predictive model across a range of threshold probabilities (0%–94%), compared to the strategies of treating all or no patients. The upper bound of 94% represents the highest probability at which the model remains clinically beneficial. **(B)** Validation cohort: The model shows a similar range of positive net benefit (0%–92%), with an upper bound of 92% indicating its consistent clinical utility.

Decision curve analysis (DCA) was performed to assess the clinical utility of the predictive model. This method calculates the net benefit of applying the model across a range of threshold probabilities, comparing it to two baseline strategies: treating all patients or treating none. The threshold probabilities, ranging from 0% to 94% in the development cohort and 0%–92% in the validation cohort, represent the minimum predicted risk at which a clinical decision (e.g., initiating treatment) would be made. The model showed a positive net benefit within these ranges, supporting its utility for patients undergoing non-cardiac, non-vascular, and non-neurosurgical surgeries (Figures 5A,B).

**FIGURE 6:**
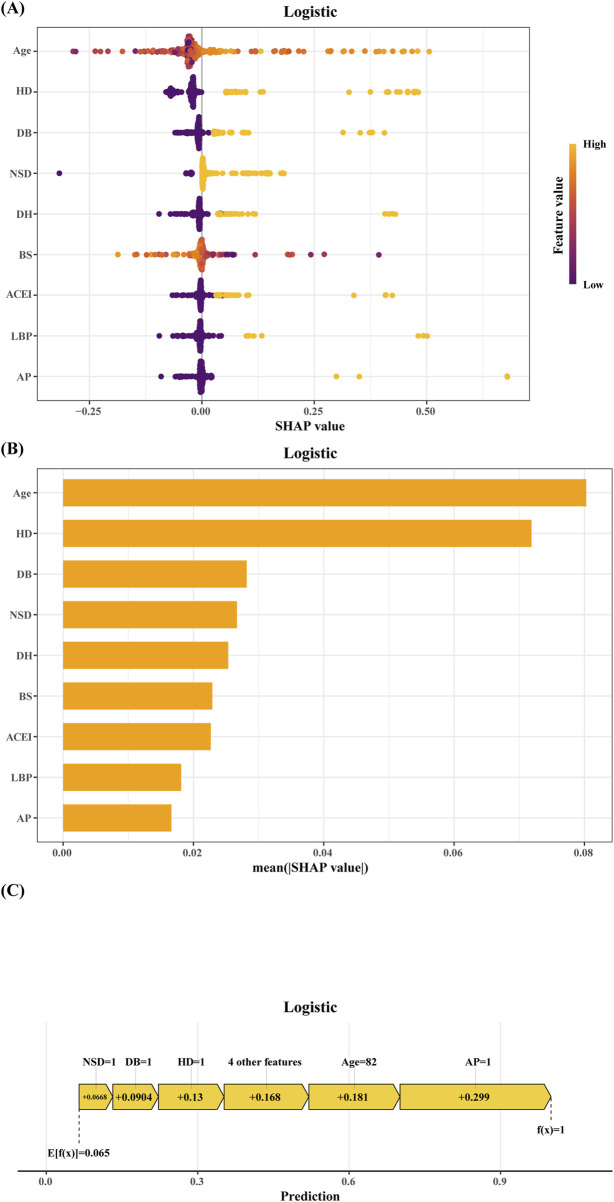
SHAP of the model: **(A)** Characteristic attributes in SHAP. The abscissa is the SHAP value, and each line denotes a feature. Higher eigenvalues are indicated by purple dots, and lower eigenvalues are indicated by yellow dots; **(B)** Feature importance ranking of the Logistic regression model; **(C)** Interpretability analysis of 1 independent samples. HD:history of cerebrovascular disease. DB: diabetes. NSD: perioperative non-steroidal drugs. DH: drinking history. BS: preoperative blood sodium level. ACEI: preoperative ACEI drugs. LBP: intraoperative MAP ≤60 mmHg for 5–10 min. AP, angina pectoris.

Figure 6A presents the SHAP (SHapley Additive exPlanations) summary plot for Logistic regression model, where the X-axis represents SHAP values (higher values indicate stronger contributions to stroke prediction). Feature magnitude is represented by a color gradient, ranging from purple (high values) to yellow (low values).

The top four predictors of stroke risk were age, a history of cerebrovascular disease, diabetes, and the use of perioperative non-steroidal anti-inflammatory drugs (NSAIDs). Advanced age, a prior history of cerebrovascular disease, diabetes, and the perioperative use of NSAIDs were strongly associated with an elevated risk of stroke. Other significant factors included a history of alcohol consumption, abnormal serum sodium levels, low usage rates of ACE inhibitors, intraoperative hypotension (mean arterial pressure [MAP] ≤ 60 mmHg for 5–10 min), and a history of angina pectoris.

These findings were further supported by the feature importance rankings in Figure 6B. The interpretability of the model was also demonstrated through individualized case analyses. Figure 6C shows SHAP force plots for a representative non-stroke patient, highlighting feature-specific contributions to the prediction.

Our thorough validation process confirmed the model’s effectiveness in clinical settings. We developed and validated a logistic regression model based on patient characteristics and stroke risk factors, employing various graphical methods for evaluation.

## Discussion

Perioperative strokes in non-cardiac and non-neurological surgery patients are relatively rare, occurring in approximately 0.1%–0.8% of patients. Notably, the incidence rises to 7% among patients older than 65 years, with many strokes remaining undetected (Lindberg and Flexman, 2021). Although less frequently diagnosed following non-cardiac, non-carotid, or non-neurological surgeries, these strokes are significant postoperative complications, contributing to increased perioperative mortality, morbidity, longer hospital stays, and higher healthcare costs (Ng et al., 2011; Lewis et al., 2019).

Preventing perioperative strokes is challenging due to complex and diverse risk factors. Common comorbidities in these patients, such as atrial fibrillation, diabetes, and hypertension, are all significant stroke risk factors (Reinert et al., 2021). Additionally, perioperative stress responses, medication adjustments, and the surgery itself can further elevate stroke risk (Vlisides and Mashour, 2016).

Clinical prediction models are essential for accurately forecasting perioperative strokes. These models, which integrate factors such as age, medical history, and surgery type, are effective at identifying high-risk patients and outperform traditional assessment methods (Goeller and Bartels, 2021). Unlike traditional statistical models, machine learning models capture complex, nonlinear relationships between risk factors, improving prediction accuracy. This is especially useful in clinical practice, where risk factors often interact in ways that are difficult for traditional models to fully account for. Developing personalized treatment plans based on these models can enhance treatment efficacy and reduce unnecessary interventions. However, the effectiveness of these treatments depends on data quality and adaptation to various clinical scenarios (Woo et al., 2021).

Applying these models in resource-limited settings remains challenging, necessitating continuous improvements to ensure their relevance in clinical practice and bridge the gap between clinical and research settings (Goeller and Bartels, 2021; Woo et al., 2021).

This study focuses on ischemic stroke and its risk factors during the perioperative period. Through statistical analysis and comprehensive evaluation, the model’s reliability in predicting perioperative stroke risk in non-cardiac surgery patients was confirmed (Goeller and Bartels, 2021; Woo et al., 2021). The model can help clinicians develop early, tailored treatment strategies.

Our model incorporates nine predictive factors (Vlisides and Moore, 2021): age, drinking history, diabetes, angina pectoris, cerebrovascular disease history, intraoperative MAP ≤60 mmHg for 5–10 min, preoperative blood sodium level, preoperative ACE inhibitor use, and perioperative NSAID use. By incorporating clinically relevant variables and their coefficients, along with the SHAP (Shapley Additive Explanations) interpretability model, the model improves interpretability. This approach enables clinicians to assess the relative importance of each predictor in perioperative stroke risk. The inclusion of SHAP further facilitates informed decision-making by offering clear insights into the contribution of each factor, thereby identifying actionable variables for intervention.

Preoperatively, stroke risk is positively correlated with advanced age, alcohol consumption, diabetes, angina, and cerebrovascular disease history (Pai et al., 2017). Additionally, low serum sodium levels and cessation of ACE inhibitors during the perioperative period increase stroke risk (Roshanov et al., 2017; Khan et al., 2021). Intraoperatively, prolonged low MAP and NSAID use are key risk factors (Pai et al., 2017; Vlisides and Moore, 2021).

Wu and Fang (2020) developed a stroke prediction model for individuals over 60, using SMOTE technology to balance the data, identifying sex, LDL cholesterol, blood glucose, hypertension, and uric acid as key predictors. (Wu and Fang, 2020). Another study involving patients over 65 years old undergoing non-cardiac surgery used various machine learning techniques to evaluate factors such as age, chest pain history, heart failure symptoms, high-risk surgeries, intraoperative blood pressure, serum creatinine levels, left ventricular ejection fraction, and perioperative transfusions to predict adverse cardio-cerebral events (Wu et al., 2023). These findings highlight the value of machine learning in risk factor analysis.

Unlike previous studies that focused on older populations or specific patient groups, our study included adult patients of all ages undergoing non-cardiac, non-vascular, and non-neurosurgical procedures, improving the generalizability of the findings. By using advanced machine learning techniques, including comprehensive imputations and integrating logistic and LASSO regression, we addressed challenges such as nonlinearity and multicollinearity, resulting in a highly interpretable and clinically relevant model. These methods, supported by previous research comparing machine learning to traditional logistic regression in prognostic modeling (Liew et al., 2022), produced exceptional AUC values of 0.864 and 0.869 in the development and validation cohorts, respectively. Internal validation confirmed the model’s robustness and its potential to guide early interventions and improve perioperative stroke prevention strategies.

This study has several limitations. First, as a single-center investigation, the findings require external validation (Riley et al., 2021). Additionally, the retrospective design introduces potential biases, such as selection bias, and limits the ability to account for changes in clinical practices over time. To mitigate these limitations, we employed random sampling, robust statistical methods, and thorough model validation to minimize bias.

The model primarily focuses on preoperative and intraoperative variables, such as age, medical history, and surgical conditions. It is designed for perioperative risk stratification and guiding postoperative management rather than preoperative decision-making. While retrospective data lacks prospective validation, it offers an efficient approach for developing and validating predictive models using large, real-world datasets. These findings provide critical insights for optimizing perioperative management and lay the groundwork for future multi-center, prospective studies to integrate preoperative predictors and enhance clinical decision-making.

## Conclusion

This study developed and validated a robust predictive model for perioperative stroke risk in non-cardiac, non-vascular, and non-neurosurgical patients. Despite its retrospective design, the model demonstrated strong discriminatory performance and clinical relevance. These findings provide a solid foundation for future multi-center, prospective studies to refine the model, incorporate additional variables, and expand its applicability to diverse patient populations.

## Data Availability

The raw data supporting the conclusions of this article will be made available by the authors, without undue reservation.

## References

[B1] Abraham J. Bartek B. Meng A. Ryan King C. Xue B. Lu C. (2023). Integrating machine learning predictions for perioperative risk management: towards an empirical design of a flexible-standardized risk assessment tool. J. Biomed. Inf. 137, 104270. 10.1016/j.jbi.2022.104270 36516944

[B2] Chahine Y. Magoon M. J. Maidu B. Del Álamo J. C. Boyle P. M. Akoum N. (2023). Machine learning and the conundrum of stroke risk prediction. Arrhythm. Electrophysiol. Rev. 12, e07. 10.15420/aer.2022.34 37427297 PMC10326666

[B3] Fernandes M. P. B. Armengol de la Hoz M. Rangasamy V. Subramaniam B. (2021). Machine learning models with preoperative risk factors and intraoperative hypotension parameters predict mortality after cardiac surgery. J. Cardiothorac. Vasc. Anesth. 35 (3), 857–865. 10.1053/j.jvca.2020.07.029 32747203

[B4] Goeller J. K. Bartels K. (2021). Improving prediction to prevent perioperative morbidity. Br. J. Anaesth. 127 (5), 671–674. 10.1016/j.bja.2021.08.004 34503833

[B5] Khan F. W. Fatima B. Lahr B. D. Greason K. L. Schaff H. V. Dearani J. A. (2021). Hyponatremia: an overlooked risk factor associated with adverse outcomes after cardiac surgery. Ann. Thorac. Surg. 112 (1), 91–98. 10.1016/j.athoracsur.2020.08.030 33080237

[B6] Lewis D. J. Al-Ghazawi S. S. Al-Robaidi K. A. Thirumala P. D. (2019). Perioperative stroke associated in-hospital morbidity and in-hospital mortality in common non-vascular non-neurological surgery. J. Clin. Neurosci. 67, 32–39. 10.1016/j.jocn.2019.06.034 31272832

[B7] Liew B. X. W. Kovacs F. M. Rügamer D. Royuela A. (2022). Machine learning *versus* logistic regression for prognostic modelling in individuals with non-specific neck pain. Eur. Spine J. 31 (8), 2082–2091. 10.1007/s00586-022-07188-w 35353221

[B8] Lindberg A. P. Flexman A. M. (2021). Perioperative stroke after non-cardiac, non-neurological surgery. BJA Educ. 21 (2), 59–65. 10.1016/j.bjae.2020.09.003 33889431 PMC7810781

[B9] Marcucci M. Chan M. T. V. Smith E. E. Absalom A. R. Devereaux P. J. (2023). Prevention of perioperative stroke in patients undergoing non-cardiac surgery. Lancet Neurol. 22 (10), 946–958. 10.1016/s1474-4422(23)00209-0 37739575

[B10] Mashour G. A. Shanks A. M. Kheterpal S. (2011). Perioperative stroke and associated mortality after noncardiac, nonneurologic surgery. Anesthesiology 114 (6), 1289–1296. 10.1097/ALN.0b013e318216e7f4 21478735

[B11] Ng J. L. Chan M. T. Gelb A. W. (2011). Perioperative stroke in noncardiac, nonneurosurgical surgery. Anesthesiology 115 (4), 879–890. 10.1097/ALN.0b013e31822e9499 21862923

[B12] Pai S. L. Wang R. D. Aniskevich S. (2017). Perioperative stroke: incidence, etiologic factors, and prevention. Minerva Anestesiol. 83 (11), 1178–1189. 10.23736/s0375-9393.17.11976-0 28901120

[B13] Reinert N. J. Patel B. M. Al-Robaidi K. Gao X. Fabio A. Jadhav A. (2021). Perioperative stroke-related mortality after non-cardiovascular, non-neurological procedures: a retrospective risk factor evaluation of common surgical comorbidities. J. Perioper. Pract. 31 (3), 80–88. 10.1177/1750458920911830 32301383

[B14] Riley R. D. Debray T. P. A. Collins G. S. Archer L. Ensor J. van Smeden M. (2021). Minimum sample size for external validation of a clinical prediction model with a binary outcome. Stat. Med. 40 (19), 4230–4251. 10.1002/sim.9025 34031906

[B15] Roshanov P. S. Rochwerg B. Patel A. Salehian O. Duceppe E. Belley-Côté E. P. (2017). Withholding *versus* continuing angiotensin-converting enzyme inhibitors or angiotensin II receptor blockers before noncardiac surgery: an analysis of the vascular events in noncardiac surgery patIents cOhort evaluatioN prospective cohort. Anesthesiology 126 (1), 16–27. 10.1097/aln.0000000000001404 27775997

[B16] Vlisides P. Mashour G. A. (2016). Perioperative stroke. Can. J. Anaesth. 63 (2), 193–204. 10.1007/s12630-015-0494-9 26391795 PMC4720532

[B17] Vlisides P. E. Moore L. E. (2021). Stroke in surgical patients. Anesthesiology 134 (3), 480–492. 10.1097/aln.0000000000003664 33411913

[B18] Woo S. H. Marhefka G. D. Cowan S. W. Ackermann L. (2021). Development and validation of a prediction model for stroke, cardiac, and mortality risk after non-cardiac surgery. J. Am. Heart Assoc. 10 (4), e018013. 10.1161/jaha.120.018013 33522252 PMC7955339

[B19] Wu X. Hu J. Zhang J. (2023). Machine learning-based model for predicting major adverse cardiovascular and cerebrovascular events in patients aged 65 years and older undergoing noncardiac surgery. BMC Geriatr. 23 (1), 819. 10.1186/s12877-023-04509-6 38062353 PMC10704781

[B20] Wu Y. Fang Y. (2020). Stroke prediction with machine learning methods among older Chinese. Int. J. Environ. Res. Public Health 17 (6), 1828. 10.3390/ijerph17061828 32178250 PMC7142983

